# Risk prediction in multicentre studies when there is confounding by cluster or informative cluster size

**DOI:** 10.1186/s12874-021-01321-x

**Published:** 2021-07-04

**Authors:** Menelaos Pavlou, Gareth Ambler, Rumana Z. Omar

**Affiliations:** Department of Statistical Science, UCL, London, UK

## Abstract

**Background:**

Clustered data arise in research when patients are clustered within larger units. Generalised Estimating Equations (GEE) and Generalised Linear Models (GLMM) can be used to provide marginal and cluster-specific inference and predictions, respectively.

**Methods:**

Confounding by Cluster (CBC) and Informative cluster size (ICS) are two complications that may arise when modelling clustered data. CBC can arise when the distribution of a predictor variable (termed ‘exposure’), varies between clusters causing confounding of the exposure-outcome relationship. ICS means that the cluster size conditional on covariates is not independent of the outcome. In both situations, standard GEE and GLMM may provide biased or misleading inference, and modifications have been proposed. However, both CBC and ICS are routinely overlooked in the context of risk prediction, and their impact on the predictive ability of the models has been little explored. We study the effect of CBC and ICS on the predictive ability of risk models for binary outcomes when GEE and GLMM are used. We examine whether two simple approaches to handle CBC and ICS, which involve adjusting for the cluster mean of the exposure and the cluster size, respectively, can improve the accuracy of predictions.

**Results:**

Both CBC and ICS can be viewed as violations of the assumptions in the standard GLMM; the random effects are correlated with exposure for CBC and cluster size for ICS. Based on these principles, we simulated data subject to CBC/ICS. The simulation studies suggested that the predictive ability of models derived from using standard GLMM and GEE ignoring CBC/ICS was affected. Marginal predictions were found to be mis-calibrated. Adjusting for the cluster-mean of the exposure or the cluster size improved calibration, discrimination and the overall predictive accuracy of marginal predictions, by explaining part of the between cluster variability. The presence of CBC/ICS did not affect the accuracy of conditional predictions. We illustrate these concepts using real data from a multicentre study with potential CBC.

**Conclusion:**

Ignoring CBC and ICS when developing prediction models for clustered data can affect the accuracy of marginal predictions. Adjusting for the cluster mean of the exposure or the cluster size can improve the predictive accuracy of marginal predictions.

**Supplementary Information:**

The online version contains supplementary material available at 10.1186/s12874-021-01321-x.

## Background

Clustered data arise in research when members are clustered within larger units. For example, patients may be clustered within health institutions, or be treated by different surgeons. In these situations, the within-cluster outcomes tend to be correlated, i.e. the outcomes for patients within a centre are more similar between them than with patients from other centres, even after accounting for their patient-specific characteristics. Ignoring this correlation may lead to biased variance estimates for the regression coefficients in a regression model, but unbiased estimates for the regression coefficients. The clustering can be accounted for with the use of Generalised Linear Mixed Models (GLMM) and Generalised Estimating Equations (GEE) which provide cluster-specific inference and marginal inference, respectively.

Confounding by Cluster (CBC) and Informative Cluster Size (ICS) are two complications that can arise when data are clustered within larger units. In observational studies, the distribution of a predictor variable (‘exposure’) often varies between clusters. Confounding by Cluster (CBC) [1–3] arises when the outcome for a member of a cluster given the exposure and other covariates is associated with the values of the exposure of other members in the cluster. In this case, the cluster is said to cause confounding of the relationship between exposure and outcome. If both the exposure and outcome are binary, CBC may arise if the proportion of exposed individuals varies considerably between clusters and this proportion relates to the between cluster variability of the outcome, i.e. the proportion of patients with the event. For example, in a study with multiple centres where patients are treated for coronary angioplasty, the patients can receive either a staged or combined procedure of angioplasty [1]. When investigating the effect of a combined procedure on the risk of post-angioplasty complications, CBC may arise when the proportion of patients who receive a combined procedure differs between centres and is also related to differences in the proportion of complications between centres. Whilst this scenario is not uncommon, it is often overlooked when developing a risk model using data that are clustered within larger units.

ICS arises when the number of observations per cluster, after accounting for the exposure and covariates, is not independent of the outcome [4, 5]. For example, surgeons who perform more operations may have better outcomes than surgeons who do fewer operations perhaps due to job-acquired skills or other reasons. Similarly, larger hospital units may have better health outcomes on average than smaller units because of readily available resources and more training opportunities for the staff. In these cases, the cluster size is said to be informative.

From a modelling point of view, both CBC and ICS have been viewed as violations of standard assumptions in a GLMM [2, 6]. In the first, the random effects are correlated with the exposure, in the second with the cluster size. Similarly, for marginal models fitted using GEE, CBC and ICS arise when the expected outcome for a particular cluster member depends on the covariates values of other members in the same cluster, and the cluster size, respectively [4, 7].

The implications of these violations on inference have been extensively studied [1–3, 8]. Methods reviewed in detail by Seaman et al. (2010) [9] have been proposed for handling data which present these complications. Nevertheless, to our knowledge, CBC and ICS are either ignored or overlooked when the target of the analysis is to predict a health outcome rather than estimating the effect of an exposure variable on the outcome. Previous authors [10, 11] briefly investigated scenarios where the random effects were correlated with one of the covariates (the definition of CBC in a mixed model) in the context of risk prediction. However, in the simulation studies in these papers it was not recognised that this scenario is a case of potential CBC. Similarly, a distortion of the usual relationship between marginal and conditional regression coefficients which was present in the empirical examples was not linked to the possibility of CBC. ICS has been less considered in the context of risk prediction, although it is a possibility when dealing with clustered data. For example, it has been suggested that there might be a volume-outcome relationship between cardiac surgical outcomes and the number of procedures performed within centres, with higher procedure volume per centre linked to better clinical outcomes [12]. However, this is typically not accounted for when developing risk models for surgical outcomes.

The issues of ICS and CBC were also briefly mentioned in recent papers, but their effect on marginal and cluster-specific predictions was not investigated in depth [13, 14]. In this work we explore whether ignoring CBC/ICS has any effect on the predictive ability of risk models and investigate whether methods that have been proposed for handling these complications, can improve the predictive accuracy of risk models.

The article is organised as follows. In the next section we set the framework for the analysis of clustered data using either a marginal or a cluster-specific approach. We discuss the categories of methods suggested for handling CBC/ICS, focusing on those methods that are best suited to model development for risk prediction. We design a simulation study to explore the effect of ignoring CBC/ICS on the predictive ability of the models as opposed to using methods that account for these issues. In the ‘Results’ section we summarise the outcomes from the simulation study and illustrate practical aspects of diagnosing and handling CBC/ICS in a multicentre study where we model the risk of death in patients with spinal metastases following surgery. We conclude with a Discussion and recommendations.

## Methods

### Analysis models for clustered data and their use for predictions

Let *Y*_*ij*_ and ***X***_*ij*_ = (*X*_*ij*, 1_, *X*_*ij*, 2_, …, *X*_*ij*, *p*_)^*T*^ denote the binary outcome and the p-dimensional vector of covariates for the *j*^*th*^ member of the *i*^*th*^ cluster, *i* = 1, …, *K*; *j* = 1, …, *N*_*i*_, where *N*_*i*_ denotes the number of members in the *i*^*th*^ cluster.

There are two main categories of methods for the analysis of clustered data, according to the type of inference/type of predictions required: cluster-specific or marginal approaches. These have been discussed extensively in the context of risk prediction [10, 13, 14], and are here summarised briefly.

### Cluster-specific models

Cluster-specific models can be specified by including cluster-specific terms for each cluster in the form of random effects which are assumed to be drawn from a distribution. Such models are known as Random Effect models or Generalised Linear Mixed Models (GLMMs). Most often the random effects are assumed to follow a Normal distribution. In this work we focus on binary outcomes modelled using logistic regression.

The commonly used logistic model with random intercepts can be written as follows:

$$ \mathrm{logit}\ \left(P\left({Y}_{ij}=1|{\boldsymbol{X}}_{ij}={\boldsymbol{x}}_{ij},{u}_i\right)\right)={\alpha}_{CS}+{u}_i+{\boldsymbol{X}}_{ij}^T\ {\boldsymbol{\beta}}_{CS}\ (1) $$

where *a*_*CS*_ and ***β***_*CS*_ are the conditional regression parameters (fixed effects), and *u*_*i*_ is the random intercept for the i*th* cluster. Usually it is assumed that ${u}_i\sim N\left(0,{\sigma}_u^2\right)$. The estimated fixed effects have cluster-specific interpretation, i.e. conditional on the random intercepts. Model (1) can be fitted using Maximum Likelihood Estimation (MLE), while Restricted MLE is used for the estimation of standard errors. Estimates of the cluster-specific random effects are obtained using *empirical Bayes estimators*.

The degree of clustering in the data can be quantified by the intra-cluster correlation coefficient (ICC), which indicates the extent of differences between patients in different clusters, not attributable to patient-level characteristics. For binary outcomes, the ICC can be estimated by $ICC=\left({\sigma}_u^2\right)/\left({\pi}^2/3+{\sigma}_u^2\ \right)$ [15]. A high ICC corresponds to a high variance $, {\sigma}_u^2$, in the distribution of random effects and larger differences in the outcome between clusters due to the effect of observed or unobserved cluster-level characteristics. Its range of possible values is between 0 and 1 with lower values indicating less variation across clusters.

Two types of predictions are typically of interest when a random intercepts model is fitted on the data in hand (the development sample), conditional and marginal predictions. Conditional predictions can be obtained using both estimates of the fixed and random effects

$$ P\left({Y}_{ij}=1|{\boldsymbol{X}}_{ij}={\boldsymbol{x}}_{ij},{u}_i\right)= logi{t}^{-1}\left({\hat{\alpha}}_{CS}+{\hat{u}}_i+{\boldsymbol{X}}_{ij}^T\ {\hat{\boldsymbol{\beta}}}_{CS.}\right) $$

and can be used to predict the risk for an individual who belongs to a cluster with a known value of the random effect. If a cluster is part of the development sample, then an estimate for its random effect would be available. The inverse logit function of the predicted probability (calculated using the estimated regression coefficients, random effects and values of predictor variables) is called the linear predictor. Conditional predictions obtained by setting *u*_*i*_ = 0 in the equation above are a special type of conditional predictions for a cluster with random effect zero, representing an ‘average-risk cluster’. Marginal predictions can be obtained by integrating over the (prior) distribution of the random effects and are suitable for an individual drawn from the population of all patients, without an assumption about the cluster they belong to [13, 16]. An approximation of the marginal regression parameters to the conditional ones provided by Zeger (1988) [17] has been seen to perform equally well and is simpler to calculate and use in practice to obtain marginal prediction, without the need of numerical integration.

### Marginal models

A marginal logistic model (not conditional on random effects) can be written as:

2 $$ \mathrm{logit}\left(P\left({Y}_{ij}=1|{\boldsymbol{X}}_{ij}={\boldsymbol{x}}_{ij}\right)\right)={\alpha}_M+{\boldsymbol{X}}_{ij}^T\ {\boldsymbol{\beta}}_M $$

where *a*_*M*_ and ***β***_*M*_ are the marginal regression coefficients. The model above can be fitted using Generalised Estimating Equations (GEE) with a suitable ‘working correlation structure’, which may lead to efficient estimates (compared to using the independence working correlation) that are also robust to the misspecification of the working correlation when data are missing completely at random (MCAR). For data clustered within centres, the exchangeable working correlation is usually appropriate. GEE can be used to obtain marginal (also known as population-average) predictions:

$$ P\left({Y}_{ij}=1|{\boldsymbol{X}}_{ij}={\boldsymbol{x}}_{ij}\right)=\mathrm{logi}{\mathrm{t}}^{-1}\left({\hat{\alpha}}_M+{\boldsymbol{X}}_{ij}^T\ {\hat{\boldsymbol{\beta}}}_M\right). $$

### Marginal versus cluster-specific predictions

Cluster-specific predictions can be obtained from the fit of a random effects model while marginal predictions can be obtained from the fit of either a random effects model or a marginal model. Cluster-specific predictions may be preferred because they are more accurate [18], as long as the random effects are estimated reasonably well. Estimates of the random effects are only applicable to clusters that are part of the model-development sample. Therefore, for clusters that are not included in the development sample, marginal predictions may be used instead.

### Relationship between marginal and cluster-specific coefficients

For linear models, marginal and conditional regression coefficients coincide due to the use of the identity link function (model-collapsibility), whereas for logistic regression they do not. Nevertheless, in the special case of logistic regression with random intercepts, the marginal regression parameters are in magnitude closer to zero than the corresponding conditional ones, and an approximate relationship between the two exists [17, 19]. The random effects model assumes that the exposure is independent of the random effects. Violation of this assumption can distort the relationship between marginal and conditional coefficients [2]. Any departures from this relationship for the estimated coefficients should trigger further investigations for the cause of these departures.

Confounding by cluster and informative cluster size are two complications likely to arise separately or even concurrently when modelling clustered data. They can both be viewed as violations of standard assumptions of both random effects and marginal models. Several modifications of GLMM and GEE have been proposed to handle data with CBC/ICS and were previously reviewed in detail [9]. Here we discuss the potential impact of ignoring CBC/ICS on the accuracy of predictions, and focus on identifying proposed methods that are appropriate for prediction.

### Confounding by cluster (CBC)

When the distribution of a cluster-varying predictor variable termed as exposure and denoted by *R* differs between clusters (for example the mean exposure or the proportion of exposed individuals), then the cluster may confound the association between exposure and outcome causing confounding by cluster (CBC). For a random intercepts model this means that the random intercepts are not independent of the exposure variable. Similarly, for a marginal model fitted using GEE, CBC arises when the expected outcome for a particular cluster member depends on the covariate values of other members of the same cluster, $E\left({Y}_{ij}|{X}_{ij}\right)\ne E\left({Y}_{ij}|{X}_{i1},{X}_{i2},\dots, {X}_{i{N}_i}\right)$[7]. Ignoring CBC in fitting a marginal or a cluster-specific model, results in bias in the estimated regression coefficients of the exposure, which may have impact on the predictive ability of the model.

Two categories of methods have been proposed for handling CBC, primarily focusing on the estimation of the ‘within-cluster’ effect of the exposure. In the first approach, the exposure variable is decomposed into the within-cluster and between-cluster components, ${R}_{ij}-{\overline{R}}_i$ and ${\overline{R}}_i$ respectively, where ${\overline{R}}_i={\sum}_{ij}{R}_{ij}/{N}_i.$ More general cases have also been considered where $\overline{R_i}$ is replaced by another cluster-level summary of *R*_*ij*_ rather than the average, for example max(*R*_*ij*_) [8, 20].

This decomposition approach can be applied both for GLMM and GEE as below:

3 $$ \mathrm{logit}\ \left(P\left({Y}_{ij}=1|{\boldsymbol{X}}_{ij}={\boldsymbol{x}}_{ij},{R}_{ij}={r}_{ij},{u}_i\right)\right)={\alpha}_{CS}+{u}_i+{\boldsymbol{X}}_{ij}^T\ {\boldsymbol{\beta}}_{CS}+\left({R}_{ij}-{\overline{R}}_i\right)\ {\gamma}_{W, CS}+{\overline{R}}_i{\gamma}_{B, CS} $$

4 $$ \mathrm{logit}\ \left(P\left({Y}_{ij}=1|{\boldsymbol{X}}_{ij}={\boldsymbol{x}}_{ij},{R}_{ij}={r}_{ij}\right)\right)={\alpha}_M+{\boldsymbol{X}}_{ij}^T\ {\boldsymbol{\beta}}_M+\left({R}_{ij}-{\overline{R}}_i\right)\ {\gamma}_{W,M}+{\overline{R}}_i{\gamma}_{B,M} $$

where *γ*_*W*, *CS*_ and *γ*_*B*, *CS*_ denote the within- and between-cluster effects, respectively (and analogously for *γ*_*W*, *M*_ and *γ*_*B*, *M*_). When CBC is not present, the within- and between-cluster effects are equal. However, they differ when there is CBC, and scientific interest most often lies on the within-cluster-effects. The possible implications of CBC on prediction are discussed below. Note that an alternative parameterization of (3) and (4) uses just *R*_*ij*_ in place of $\left({R}_{ij}-{\overline{R}}_i\right)$.

The second category of methods is based on ‘Conditional Maximum Likelihood’ estimation (CML), where all cluster differences in the outcome and exposure are removed by conditioning out all cluster-level information. The result is unbiased inference about the within-cluster effect of the exposure. CML is appealing because it protects against confounding and requires weaker assumptions than the decomposition approach [9]. However, it cannot estimate the effect of any cluster-constant variables and is generally less efficient than the decomposition approach described above.

Among these two approaches, the decomposition method is the one most suited for prediction because it allows the estimation of the between-cluster effect of the exposure $, {\overline{R}}_i$. This can itself be of scientific interest but importantly can also be a predictor of the outcome on its own right [2]. When using a GLMM as in (3) above, adjustment for the cluster-level covariate ${\overline{R}}_i$ (as with any other cluster-level variable that associates with the outcome) can explain at least part of the between-cluster variability, thus reducing the estimated variance of ${\sigma}_u^2$ compared to the standard GLMM that does not adjust for ${\overline{R}}_i$. This can have important implications when CBC is present. Suppose that predictions are sought for individuals who belong in new clusters (not part of the development dataset). In this case, estimates of the random intercepts for the new centres are not available. Marginal predictions obtained from models (3) and (4) are expected to be more accurate than the corresponding models which do not adjust for ${\overline{R}}_i$. CML does not offer this advantage as it does not allow estimation of effects for cluster-level variables, nevertheless it can still be a useful tool to assess the presence of CBC.

### Detecting CBC

Several approaches have been proposed for formally or informally testing for CBC [1]. Necessary conditions for CBC are the between cluster-variation in the distribution of the covariate and in the distribution of the outcome given the covariate. As an informal diagnostic for the presence of CBC, the cluster-specific coefficient estimates (from a standard GLMM under non-ICS or Conditional ML in general) and the population-average estimates (from GEE with independence working correlation) are compared. In the absence of CBC, the marginal regression coefficients are in magnitude closer to zero than the corresponding conditional ones [2]. A distortion of this relationship for the estimated marginal and conditional coefficients is suggestive of CBC [8].

### Informative cluster size (ICS)

ICS may arise when the number of members per cluster varies and the outcome is not independent of the cluster size, even after adjusting for covariates, *E*(*Y*_*ij*_| *X*_*ij*_) ≠ *E*(*Y*_*ij*_| *X*_*ij*_, *N*_*i*_) [4, 21]. In dental studies, the effect of lifestyle and other factors on periodontal disease (a gum disease the severity of which is measured by the gum detachment) may be of interest. The number of teeth per patient may vary because of lifestyle exposures or genetic characteristics, may have already lost some of their teeth to the disease. So, the number of teeth within a patient may not be independent of the disease status. In studies investigating the association between the volume of procedures carried out per surgeon, the performance of surgeons performing a specific operation assessed by the proportion of successful operations, may be associated with the number of operations performed [22].

Both the standard GLMM and models based on GEE are affected when the cluster size is informative. For GLMM previous authors primarily focused on models with random intercept terms only, and not random slope terms. Depending on the scientific question and the type of model used (marginal versus conditional) various methods have been used for dealing with ICS. In most cases considered in the literature, the target of the analysis has been the unbiased estimation of the effect of a cluster-constant or a cluster-varying exposure (or predictor for risk model). For GEE, various weighting schemes depending on the type of the exposure (cluster-constant or cluster varying) have been proposed, all of which require that GEE be fitted with the independence working correlation [9]. In the simplest scenario with ICS, the predictors are assumed to be either cluster-constant or their distribution be independent of the cluster size. In this case, the contribution of the members who belong to the same cluster in the estimating equations is divided by the number of members in the cluster to provide unbiased inference [4]. For more complex schemes, e.g. cluster-varying predictor whose distribution is not independent of the cluster sizes, different types weights have been used [23]. For GLMM, where the random intercepts are assumed to be independent of the predictors but non-independent of the cluster size, the standard GLMM results in unbiased estimation of covariate effects but biased estimation of the intercept term. The joint modelling approach, where a separate model for the cluster-size (e.g. continuation ratio joint model) having shared random effects with the main outcome model has been proposed for these cases [24]. However, this has been of limited use due to its complexity, since unbiased estimation of the covariate effects can be obtained using simpler methods.

When the aim of the analysis is the unbiased estimation of the effects of predictors, adjusting for cluster size (or a function of cluster-size) in a regression model is generally not appropriate because researchers are interested in the overall effect of a predictor, rather than conditional on cluster size, or because the cluster size lies in the causal pathway between outcome and exposure [9]. However, when the target of the analysis is prediction rather than estimation of predictor exposure effects, the reasons for avoiding the inclusion of cluster size as a covariate become less relevant. In this case, interest also lies directly on the effect of the cluster size on the outcome and its role as a potential predictor.

### Detecting ICS

A straightforward approach to detect ICS, and relevant in the context of risk prediction, is to fit a regression model for the outcome, such as (1) or (2) above, but including *N* (and/or functions of *N* such *N*^2^, log(*N*)), alongside the other predictor variables and test whether the effect of *N* is zero [9]. A significant effect would indicate that the cluster size is informative, and therefore including cluster size (or functions of it) in the developed risk model is likely to result in improved marginal predictions. Other approaches for detecting ICS, which do not require specifying a functional form for the dependence of the outcome on cluster size, after accounting for other predictor variables [25]. Exploratory analysis using summary statistics on the prevalence of outcome across clusters of different sizes may also provide some evidence about the possibility of ICS.

### Handling clustered data with ICS/CBC in the context of risk prediction

CBC and ICS may arise separately but in more complex scenarios combinations of ICS and CBC are also possible. In the context of risk prediction, adjusting for functions of cluster size and/or the mean of the exposure is recommended, as below:

5 $$ \mathrm{logit}\ \left(P\left({Y}_{ij}=1|{\boldsymbol{X}}_{ij}{R}_{ij},{N}_i,{u}_i\right)\right)={\alpha}_{CS}+{u}_i+{\boldsymbol{X}}_{ij}^T\ {\boldsymbol{\beta}}_{CS}+{\gamma}_{1, CS}{R}_{ij}+{\gamma}_{2, CS}\ f\left({\overline{R}}_i\right)+{\delta}_{CS}\ g\left({N}_i\right) $$

6 $$ \mathrm{logit}\ \left(P\left({Y}_{ij}=1|{\boldsymbol{X}}_{ij}{R}_{ij},{N}_i,\right)\right)={\alpha}_M+{\boldsymbol{X}}_{ij}^T\ {\boldsymbol{\beta}}_M+{\gamma}_{1,M}{R}_{ij}+{\gamma}_{2,M}f\left({\overline{R}}_i\right)+{\delta}_M\ g\left({N}_i\right) $$

In the simplest of scenarios where either ICS or CBC is present, adjusting for either *g*(*N*) = *N* or $f\left(\overline{R}\right)=\overline{R}$ in the regression model for the outcome is sufficient. In more general scenarios the outcome may depend on *N* or $\overline{R}$ in a non-linear manner. In these scenarios, suitable functions of *N* and $\overline{R}$ may be chosen instead, provided that there is a large enough sample size to unveil these relationships without inducing model overfitting. For example, if a U-shape is suspected for the relationship between the cluster size and the outcome, polynomial terms of *N* can be included in the model. Alternatively non-linearity may be more flexibly accommodated by the inclusion of dummy variables based on a categorisation of cluster sizes into a number of categories that are likely to capture the non-linearity. Care should be taken with this latter approach when the number of events is small, because it increases the number of regression coefficients to be estimated and the danger of model-overfitting.

In practice, it is possible that both ICS and CBC are present and adjustment for both *N* and $\overline{R}$ will be necessary. If the mechanisms giving rise to CBC and ICS are closely linked, adjusting for one of *N* or $\overline{R}$ in a regression model could remove or reduce the need for adjusting for the other. For example, suppose that the proportion of exposed individuals varies across clusters and there is CBC. We assume further than the cluster sizes vary and are related to the outcome, and also that the higher the proportion of exposed individuals the smaller the size of the cluster. For a model that adjusts neither for *N* nor for $\overline{R}$ there will be both ICS and CBC. However, adjusting for $\overline{R}$ alongside the other predictors to handle CBC may also make the cluster size non-informative as the two are so closely related, and vice versa; adjusting for *N*, may remove CBC.

### Simulation study

#### Aims

The aims of the simulation study are to:

1) Assess how the predictive performance of the basic model (B) that ignores CBC/ICS is affected when CBC/ICS are present, and whether it differs depending on the type of model (cluster-specific/marginal) and type of predictions (cluster -specific/marginal).

2) Assess whether simple adjustments to account for CBC/ICS when they are present can also improve the predictive performance of risk models.

#### Simulation parameters

Clustered data are simulated considering the following two parameters: the presence of CBC/ICS and the degree of clustering in the data:

- Complication in the data: None, CBC or ICS
- Degree of clustering: ICC= 0.1 or 0.2.

We consider cluster-specific and marginal models

- Random effects models with random intercepts for the cluster (GLMM) which can provide either conditional or marginal predictions
- Marginal models fitted using Generalised Estimating Equations with exchangeable working correlation (GEE) and marginal model fitted by MLE or equivalently GEE with independence working correlation (IEE) which can only provide marginal predictions.

#### Adjustment for CBC/ICS

Depending on whether we wish to handle ICS/CBC we consider the following three models

- A basic model that adjusts for a set of predictors ***X*** and the exposure variable *R*, ignoring the possible presence of CBC/ICS (‘Basic’ or just ‘B’ for the figures)
- The basic model with additional adjustment for the cluster-mean of the exposure (‘ $\overline{R}$ ’)
- The basic model with additional adjustment for the cluster size (‘N’).

#### Measures of predictive performance

The cluster-specific and marginal models are fitted in training datasets generated under a given data generating mechanism (see below). We examine the predictive performance of the models for marginal and conditional predictions in terms of calibration, discrimination, and overall predictive accuracy on separate validation datasets, generated under the same mechanism. Calibration in the large and calibration slope can be estimated by fitting two separate regression models to the validation data. Calibration slope is the slope term in a logistic regression model where the estimated linear predictor is regressed on the outcome. Calibration in the large can be obtained using a logistic regression model with a single intercept term, and the estimated linear predictor included as an offset term (i.e. its regression coefficient is fixed to the value 1). The calibration in the large is the estimated intercept. A value of 0 for the calibration in the large, meaning that the average predicted probability is equal to the observed proportion of events, and a value of 1 for the calibration slope, correspond to a perfectly calibrated model. A calibration slope < 1 is an indicator of model-overfitting, suggesting that shrinkage of the regression coefficients may be necessary [26]. Model discrimination is assessed using the C-Statistic which takes values 0.5 to 1, with higher values indicating higher discriminatory ability. Overall predictive accuracy can be assessed using either the Root Predictive Mean Square Error (RPMSE) or the Brier Score. The Root Predictive Mean Square error is defined as the square root of the average of the squared differences between the true and estimated probabilities across patients. Similarly, the Brier Score is defined as the square root of the average of the squared differences between the outcome and the estimated probabilities across patients.

### Data generating mechanisms

Correlated clustered outcomes are generated using an underlying random effects model with random intercepts for the cluster. The random intercepts are allowed to be correlated with an exposure variable and/or the number of members in the cluster to induce CBC/ICS, respectively [3].

The simulation steps 1–6 generate clustered training datasets with CBC and/or ICS.

1. Generate three correlated normal variables *Z*_*ui*_, *Z*_*vi*_, *Z*_*wi*_ *i* = 1, …, *K* from a multivariate normal distribution with mean zero, variance one and pairwise correlation *ρ*_*uv*_, *ρ*_*uw*_, *ρ*_*vw*_ ≥ 0, where *K* is the number of clusters. By design, a non-zero pairwise correlation between *Z*_*u*_ and *Z*_*v*_ will induce CBC and a non-zero correlation between *Z*_*u*_ and *Z*_*w*_ will induce ICS (see steps 3, 5 and 6 below).

2. Generate a random effect for cluster, ${u}_i={\sigma}_u^2{Z}_{ui}\sim N\left(0,{\sigma}_u^2\right)$, where ${\sigma}_u^2>0$ reflects the degree of clustering, with an $ICC=\frac{\sigma_u^2}{\pi^2/3+{\sigma}_u^2}$.

3. Generate cluster sizes, *N*_*i*_, for the *i*^*th*^ cluster from a Poisson distribution:

$$ {N}_i\sim Poisson\left(\mathit{\exp}\left({a}_0+{a}_1{w}_i\right)+5\right),i=1,\dots, K $$

where $$ {w}_i={\sigma}_w^2{Z}_{wi}\sim N\left(0,{\sigma}_w^2\right) $$.

3a. For non-ICS: ${\sigma}_w^2=0$, so *N* is independent of *u*.

3b. For ICS: ${\sigma}_w^2>0$, and *ρ*_*uw*_ > 0, so *N* is not independent of *u* and hence ICS is present.

4. Generate predictors for the *i*^*th*^ cluster that are independent of *N*_*i*_ and *U*_*i*_: three continuous predictors from a Normal distribution with mean zero and variance 0.3^2^, 0.4^2^ and 0.5^2^ respectively, and two binary predictors with prevalence 0.1 and 0.2, respectively. We let ***X***_*ij*_ denote the vector of covariate values for the *j*^*th*^ member of the *i*^*th*^ cluster.

5. Generate the binary exposure

$$ {R}_{ij}\sim \mathrm{Bernoulli}\left(\mathrm{logi}{\mathrm{t}}^{-1}\left({\gamma}_0+{v}_i\right)\right),=1,\dots, K,j=1,..,{N}_i $$

where $$ {v}_i={\sigma}_v^2{Z}_{vi}\sim N\left(0,{\sigma}_v^2\right) $$ .

5a. For no CBC: ${\sigma}_v^2=0$, so *R* is independent of *u*. 5b. For CBC: ${\sigma}_v^2>0$ so *R* is not independent of *u* and hence CBC is present.

6. Generate binary outcomes from a random intercepts model with linear predictor

$$ {\eta}_{ij}={\beta}_0+{\left({\boldsymbol{X}}_{ij},{R}_{ij}\right)}^T{\boldsymbol{\beta}}_1+{u}_i,\mathrm{and}\ {Y}_{ij}\sim \mathrm{Bernoulli}\left(\mathrm{logi}{\mathrm{t}}^{-1}\left({\eta}_{ij}\right)\right),i=1,\dots, K,j=1,..,{N}_i $$

The three random effects, *u*, *v*, and *w* are used to regulate the degree/presence of clustering, CBC and ICS, respectively. By setting values for the correlations *ρ*_*uv*_, *ρ*_*uw*,_ *ρ*_*vw*_ and the variances ${\sigma}_v^2$ and ${\sigma}_w^2,$ generation of data with CBC and/or ICS can be flexibly accommodated. If *u* and *v* are uncorrelated (*ρ*_*uv*_ = 0) or ${\sigma}_v^2=0$ then there is no CBC. If *u* and *w* are uncorrelated (*ρ*_*uw*_ = 0) or ${\sigma}_w^2=0$ there is no ICS. If both *ρ*_*uv*_, *ρ*_*uw*_ >0 and ${\sigma}_v^2,{\sigma}_w^2>0$ there is both ICS and CBC arising from either two independent mechanisms (if *ρ*_*vw*_ = 0) or from two related mechanisms (if *ρ*_*vw*_ > 0).

Validation data are generated in an analogous manner. For a given training dataset, the same set of random effects is used to generate the corresponding validation dataset with the same number of clusters, and thus, both cluster-specific and marginal predictions can be obtained. For cluster-specific predictions, the covariate values of the validation dataset and both the estimates of the fixed and the random effects from the fit of the model are used, corresponding to a prediction for a new member of an existing cluster. For marginal predictions, the covariate values in the validation data and the estimated marginal regression coefficients only are used. For non-ICS in step 3, we generate larger cluster sizes (for the performance measures to be calculated accurately) for the validation data from a Poisson distribution with mean *λ* = exp(*α*), *α* ∼ Normal(5.7,0.3^2^) that is unrelated to *u* or *v*. For ICS, we generate cluster sizes from the same distribution as in step 3b.

When ${\sigma}_u^2>0$ there is a variability in the outcomes between clusters, as reflected by the random intercept *u*. We consider two scenarios of clustering taking ${\sigma}_u^2=0.82$ and 0.37 which correspond to ICCs of 0.2 and 0.1, respectively. We also take ${\sigma}_v^2=$ ${\sigma}_u^2$ and ${\sigma}_w^2=$ ${\sigma}_u^2/2$.

The proportion of between-cluster variance that is either due to differences in the proportion of individuals with the exposure or the cluster size can be controlled by *ρ*_*uv*_ and *ρ*_*uw*_. If either *ρ*_*uv*_ > 0 or *ρ*_*uw*_ > 0, the random intercept terms are associated either the cluster mean of the exposure or the clusters size, inducing CBC or ICS. Thus, the between-cluster variability is not entirely due to unobserved characteristics, but it can be partly explained by two relevant observed cluster-level characteristics: the cluster size and the proportion of members with the exposure per cluster. We use *ρ*_*uv*_ = 0.70 or *ρ*_*uw*_ = 0.70 which correspond to approximately half of the between-cluster variability ${\sigma}_u^2$ being due to either CBC or ICS. We also consider a scenario with both ICS and CBC, where *ρ*_*vw*_ = 0 for the mechanisms that cause ICS and CBC to be independent or *ρ*_*vw*_ = 0.5, for the mechanisms that cause CBC and ICS to be related. The R code for the simulation studies is provided in Supplementary Material 2.

## Results

### Simulation

We simulated 500 training and validation datasets. The ICC was initially fixed to 0.2 corresponding to true variance of the random intercepts of ${\sigma}_{u, true}^2=0.82$. Each training dataset consisted of 30 clusters. In step 3 we chose *a*_0_ = 4.5 and *a*_1_ = 1 corresponding to an average cluster size of approximately 100. In step 5, we chose *γ*_0_ = logit(0.4) so the prevalence of the exposure was approximately 40%. The intercept term was set to − 1.5 and all regression coefficients to 1, which correspond to an outcome prevalence of 40%, for both training and validation data. Four different scenarios were mainly considered depending on whether ICS/CBC were present:

S1: Non-ICS and no-CBC (‘NONE’),

S2: CBC and non-ICS (‘CBC’),

S3: ICS and no-CBC (‘ICS’).

S4: Both ICS and CBC (‘ICS + CBC’) caused by independent or related mechanisms

Below we refer to results and present Figures for ICC = 0.2. Results for ICC = 0.1 were analogous and are presented in the Supplementary Material 1.

### Aim 1: assessing the predictive performance of the basic model which ignores CBC/ICS

**Fig. 1:**
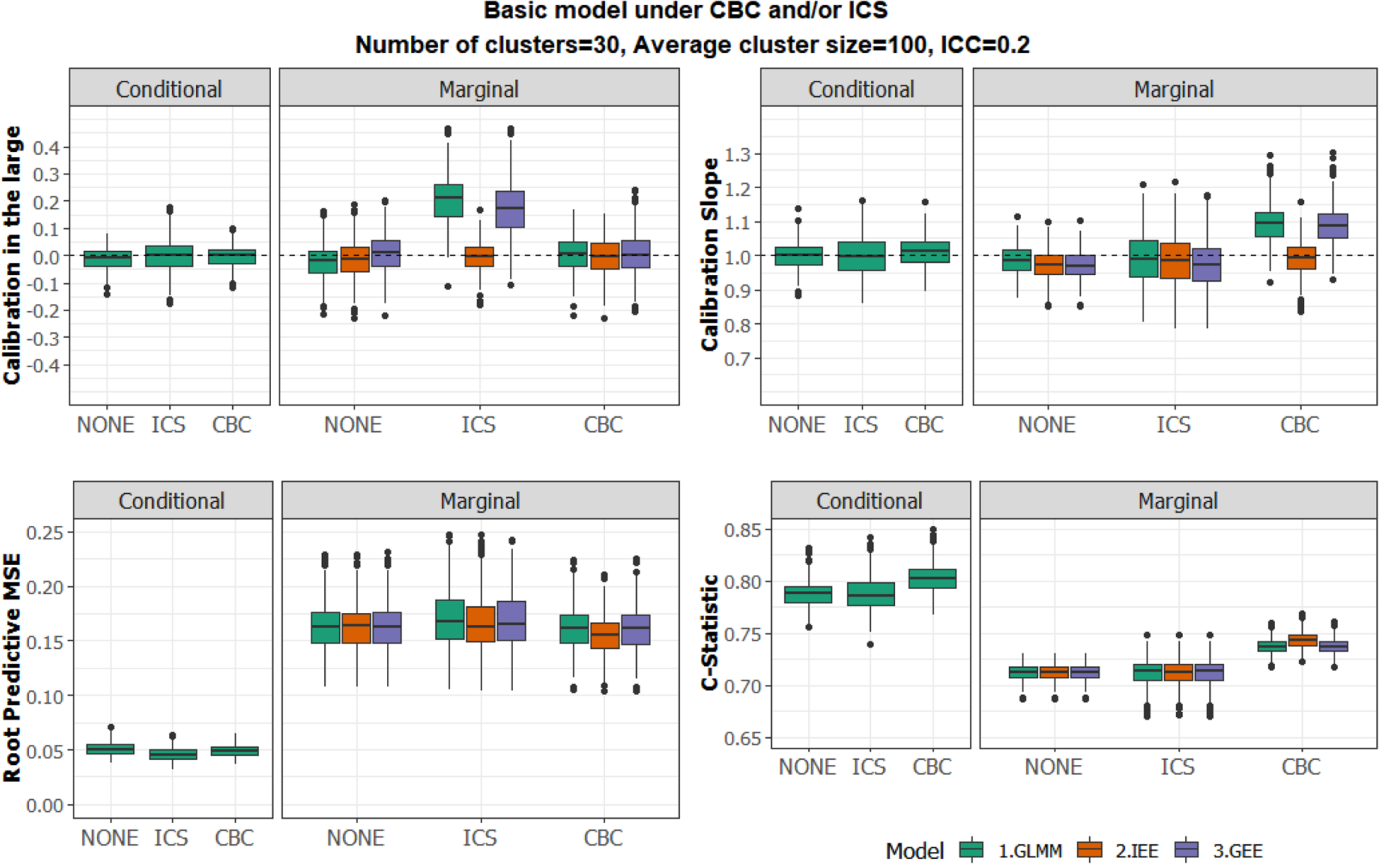
Comparison of the different fitting methods (GLMM, IEE, GEE with exchangeable correlation) for the Basic model, under different assumptions about the presence of CBC and ICS. ICC = 0.2. Performance measures from top left to bottom right: Calibration Intercept, Calibration slope, Root Predictive MSE and C-Statistic. Conditional (left Panel) and Marginal (right panel) predictions are shown for each measure

We firstly fitted the Basic model (i.e. not handling CBC and ICS when present) to assess its performance for all scenarios. The assumption of non-ICS and no-CBC holds true for S1 but not for S2-S4. In Fig. 1 we examine how the presence of CBC/ICS (S2-S3) affects the accuracy of predictions from the Basic Model, in comparison to their accuracy when CBC/ICS are absent (S1).

The predictive performance of the Basic model, fitted by all three methods was to some extent affected for S2-S3, in comparison to S1, for both marginal and conditional predictions.

#### Marginal predictions

In the presence of ICS/CBC there was evidence of model miscalibration, with the marginal predictions from GLMM and GEE being more affected than IEE. In particular, when there was ICS, the average calibration in the large for marginal predictions was 0.20 and 0.17 [these are minor typos not corrected in last revision]for GLMM and GEE, respectively, suggesting that the predictions were consistently low. When there was CBC, the average calibration slope was slightly high, 1.10 for GLMM and 1.09 for GEE, suggesting that the range of predictions was slightly narrow. Notably, marginal predictions form IEE were well calibrated in all three scenarios. The C-statistic and the RPMSE were similar for all three scenarios.

#### Conditional predictions

The C-statistic and the RPMSE were similar for all three scenarios. Letting ${\overline{\hat{\sigma}}}_{u,S}^2(B)$ denote the average of the estimated variances of the random intercepts for Basic model (*B*) under scenario *s* S1, S2, S3 over the simulated datasets, we obtained ${\overline{\hat{\sigma}}}_{u,S1}^2(B)=0.77,{\overline{\hat{\sigma}}}_{u,S2}^2(B)=0.75,$ and ${\overline{\hat{\sigma}}}_{u,S3}^2(B)=0.74,$ corresponding to an estimated ICC of approximately 0.19.

### Aim 2: assessment of improvement after accounting for CBC and/or ICS

**Fig. 2:**
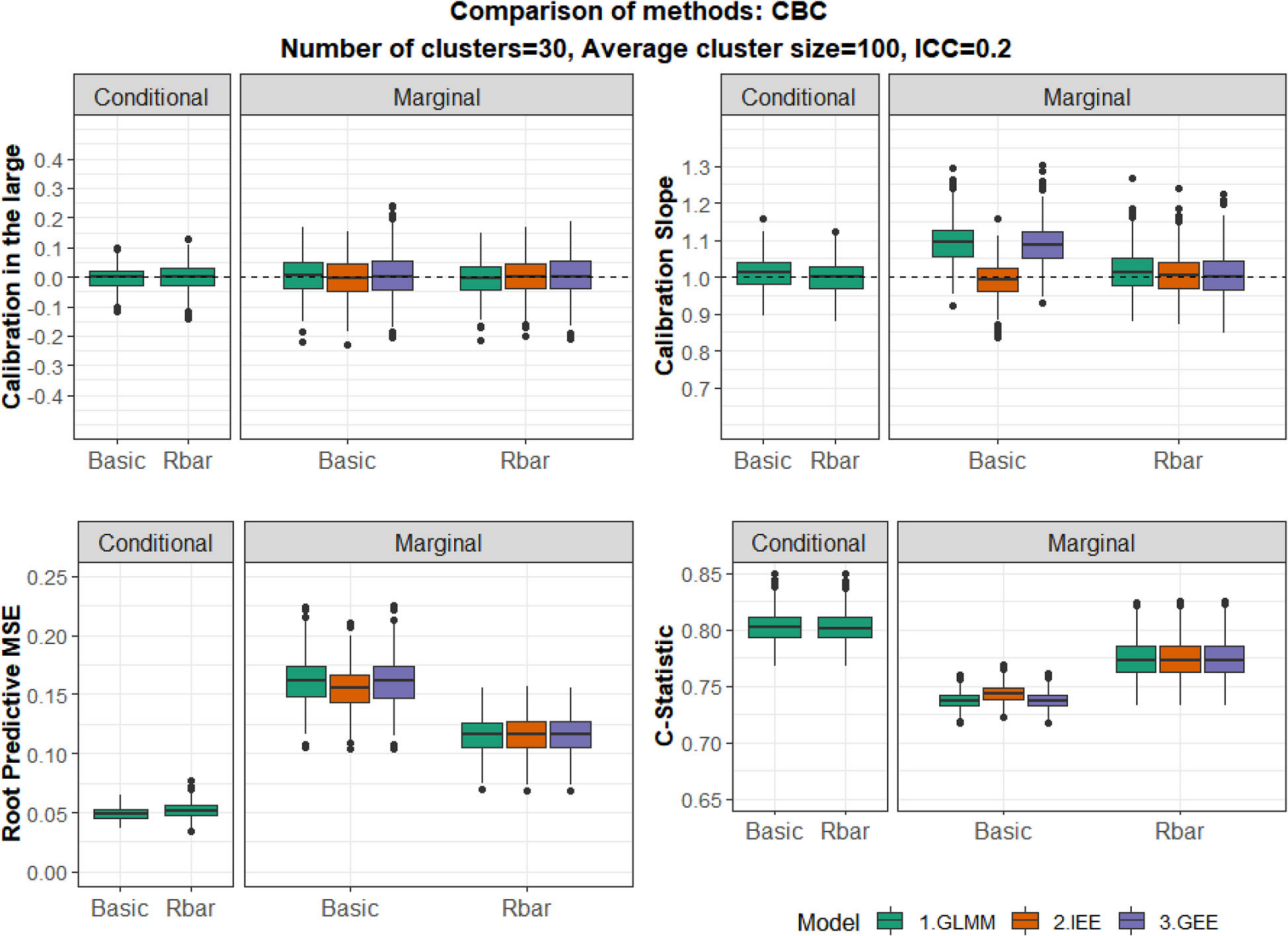
Confounding by Cluster (CBC). Comparison of Basic models (‘Basic’) and models also adjusting for the cluster mean of the exposure (‘Rbar’) fitted by three methods (GLMM, IEE, and GEE with exchangeable correlation.). ICC = 0.2. Performance measures from top left to bottom right: Calibration Intercept, Calibration slope, RPMSE and C-statistic. Conditional (left Panel) and Marginal (right panel) predictions are shown for each measure

**Fig. 3:**
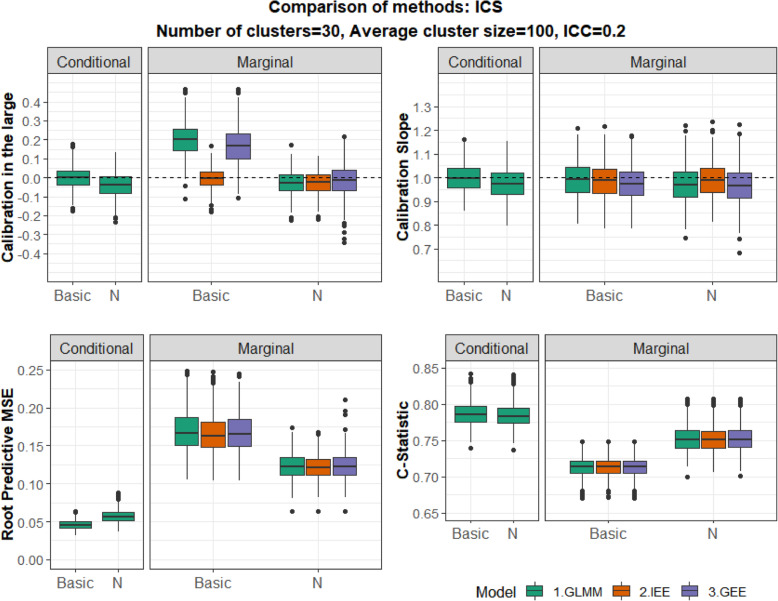
Informative Cluster size (ICS). Comparison of Basic models (‘Basic’) and models adjusting for the cluster size (‘N’) fitted by three Methods. ICC = 0.2. Performance measures from top left to bottom right: Calibration Intercept, Calibration slope, RPMSE and C-statistic. Conditional (left Panel) and Marginal (right panel) predictions are shown

We then focused on Scenarios 2, 3 and 4 where either CBC or ICS are present. In addition to the Basic model (B), we fitted models that also adjust for the cluster mean of the exposure ($B+\overline{R}$) or the cluster size (*B* + *N*) or both ($B+\overline{R}+N$). We explored whether accounting for CBC/ICS can improve the accuracy of marginal predictions in comparison to the Basic model and address the miscalibration issues. Results for S2 and S3 are presented in Figs. 2 and 3.

#### Scenario 2: CBC

We initially focus on marginal predictions. Adjusting for the cluster mean of the exposure (Fig. 2) addressed the mild miscalibration problem for the calibration slope. However, the most important finding is the improvement in the marginal predictions in terms of overall accuracy (RPMSE) and discrimination (C-Statistic) after adjusting for $\overline{R},$ in comparison to the Basic model. This can be attributed to the fact that the adjustment for the cluster-level predictor $\overline{R}$ explains a portion of the between cluster-variability in the outcome that was previously unexplained. Indeed, after adjusting for $\overline{R}$ the average of the estimated variance of the random intercepts was ${\overline{\hat{\sigma}}}_{u,S2}^2\left(B+\overline{R}\right)=0.39$ corresponding to an ICC of approximately 0.11, which is substantially lower than the estimated ICC of 0.19 for the Basic model. For conditional predictions the performances of the Basic model and the model adjusting for $\overline{R}$ are very similar. An example of the effect of CBC on the regression coefficients in models with and without adjustment for $\overline{R}$ is shown in the Supplementary Material 1.

#### Scenario 3: ICS

The results for ICS (Fig. 3) are analogous to Scenario 2 and can be interpreted in a similar manner. The minor miscalibration problem seen in the calibration in the large of marginal predictions obtained from the Basic model is addressed after adjusting for cluster size. The overall predictive accuracy and discrimination of marginal predictions are improved compared to the Basic model. In terms of conditional predictions, the performance of the Basic model and the model adjusting for cluster size are very similar.

#### Scenario 4: ICS and CBC

In practice, ICS and CBC can arise concurrently, either through two related mechanisms (for example when clusters with lower proportion of exposed individuals, also have smaller size) or through two independent mechanisms. Adjusting for both *N* and $\overline{R}$ improves the predictive ability of marginal predictions more than adjusting only for either *N* or $\overline{R}.$ This improvement, as expected, is more pronounced when ICS and CBC arise through independent mechanisms (Figures S1 and S2 in Supplementary Material 1).

### Real data illustration

Surgery is used to improve pain and maintain ambulation in patients with spinal tumour metastases. There is a considerable risk of complications associated with the surgical procedure and often the type of operation is also an individualised patient decision. Data from a multicentre study of 17 surgical centres in Europe, Asia and United States including 1179 patients with spinal metastases, who underwent surgery were available. The number of patients per centre varied between 19 and 194 with an average of 69. The average age at the time of surgery was 61 years (SD = 12.5) and 681 of the patients were men (58%).

In this illustration we aim to develop a risk model for the short-term risk of death (within a year from the date of surgery) using 4 key pre-operative factors of the patients at the time of surgery: *secondary tumour type* (renal-baseline, breast, lung, prostate, gastric/sarcoma, other), ASA physical status classification system (score 0-baseline, 1, 2 and 3/4) with 0 being best and 4 being the worst and bone metastases (0-baseline, 1–2 sites, or more than 2 sites). Death was treated as a binary outcome and modelled using logistic regression. The categorical predictors were modelled using dummy variables giving rise to 11 regression coefficients including the intercept term, and there were 424 death events.

Since data were available from 17 participating centres, we considered the random intercepts model (GLMM) as the appropriate cluster-specific model and GEE with independence correlation structure (IEE) as a population-average approach.

**Fig. 4:**
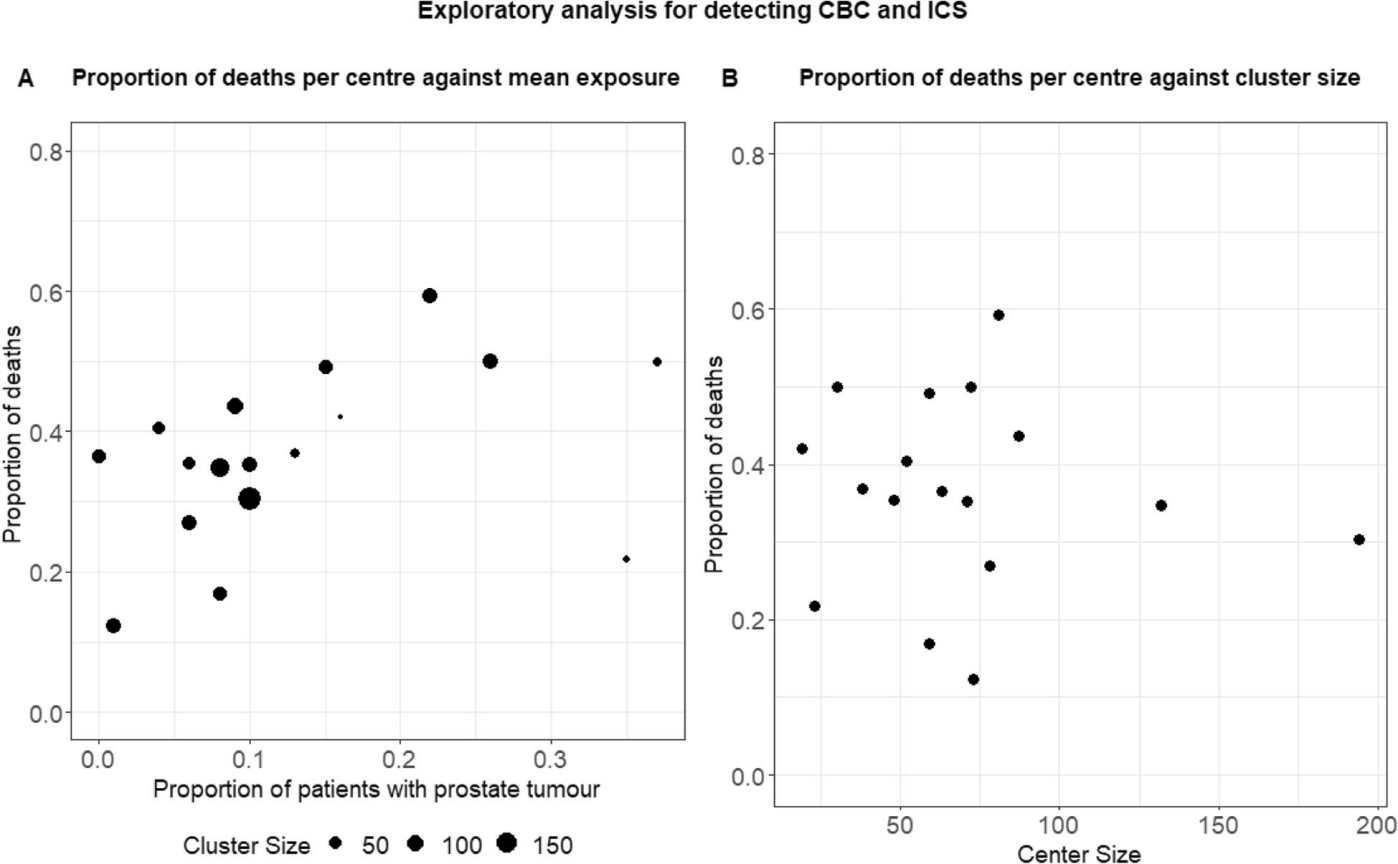
Exploratory summary statistics for CBC/ICS in the Real Data illustration. Proportion of deaths per centre against **A**) the proportion of patients with Secondary Prostate Tumour per centre and **B**) the number of patients per centre

There was considerable variability in the proportion of events and the proportion of patients with Secondary Prostate Tumour (SPT) across centres. The proportion of patients within a centre that had a SPT (exposure) ranged from 0 to 0.37 (median 0.1), while the proportion of deaths varied from 0.12 to 0.59 (median 0.37). These two conditions together suggest that confounding by cluster is a possibility in these data. In Fig. 4 we show the proportion of deaths per centre against, a) the proportion of patients with SPT and b) the number of patients per centre. We observe that the proportion of deaths per centre tends to increase with an increasing proportion of patients with SPT (with the exception of two small centres), suggesting that CBC might be present. We note that the proportion of men did not differ substantially between centres and thus the variation in SPT across centres cannot be attributed to gender imbalance between centres. A reason for the variation in the proportion of exposed individuals could be that specific centres are specialised in treating patients with specific tumour types. Another reason could be that in certain areas, patients are more susceptible to specific tumour types than to others. On the other hand, the proportion of deaths per centre does not seem to be associated with the centre size in this exploratory analysis, and therefore it does not seem likely that the centre size is informative.

We initially fitted marginal and cluster-specific models using the IEE and the GLMM, respectively, and obtained estimates for the fixed and the random effects for each method. The estimated variance of the random effects was 0.43 corresponding to an ICC of 0.12. We then compared the regression coefficients based on marginal and cluster-specific models. We observed that there was a distortion of the usual relationship between marginal and conditional coefficients for the variable SPT. The cluster-specific coefficient for SPT from conditional ML was 0.59 (SE = 0.25), from the GLMM 0.64 (SE = 0.25), closer to zero than the estimated coefficient of 0.80 (SE = 0.29) for IEE. This is suggestive of CBC.

We then adjusted for the proportion of patients in each cluster with SPT $, \overline{R},$ both for IEE and GLMM. The estimated coefficients for the within-cluster effect of *R* from IEE were 0.53 (SE = 0.254) and from GLMM 0.59 (SE = 0.25), both very close to the estimate from conditional ML. The between-cluster effect of *R* was 5.15 (SE = 1.64; *p* < 0.01) and 5.81 (SE = 1.96; *p* < 0.01), for GLMM and IEE, respectively, confirming its role as a cluster-level predictor in the risk model. Indeed, the estimated ICC after adjusting for $\overline{R}$ dropped to 0.08, confirming that that part of the between-cluster variability was explained by $\overline{R}.$ We also adjusted for cluster size in the GLMM to test for informative cluster size. The effect of cluster size was not statistically significant (*p* = 0.24).

In internal validation using bootstrapping, the marginal predictions from IEE and a model that adjusts for $\overline{R}$, were more accurate (C-statistic = 0.68 and Brier Score = 0.2056) than from a model that does not adjust for $\overline{R}$ (C-statistic = 0.65 and Brier Score = 0.2112). The corresponding estimates for conditional predictions obtained from the standard GLMM ignoring CBC were C-statistic = 0.69 and Brier Score = 0.1844. Conditional predictions after accounting for CBC were very similar in accuracy (C-statistic = 0.69 and Brier Score = 0.1863). These results confirm the conclusions of the simulation study.

## Discussion

Accounting for the clustered feature of the data when developing prediction models from multicentre studies can result in more accurate cluster-specific predictions, in comparison to approaches that ignore clustering [27]. However, this opportunity is often missed, as the overwhelming majority of prediction models developed on clustered data actually ignore clustering [28]. This is partly because the degree of clustering is small, and partly due to the higher complexity of the methods to develop a model for clustered data. Also, part of the reason is that cluster-specific predictions are typically applicable to clusters which are part of the model-development sample and for which the estimated random effects are known. For patients from clusters that do not form part of the development sample, marginal predictions may be used instead.

ICS and CBC are two complications that may be encountered when dealing with clustered data. ICS does not arise when the cluster size is constant, while CBC does not arise when the predictor of interest is either cluster-constant or mean-balanced across clusters. Previous research focused on the adaptation of the standard GLMM and standard GEE to provide unbiased estimation for exposure effects under CBC or ICS, and various methods have been proposed. In this paper we studied the effect of CBC and ICS on the predictive ability of models developed using clustered data. We have shown that ignoring these complications can affect the predictive accuracy of marginal predictions.

We have identified two simple approaches for handling CBC and ICS that are relevant when developing prediction models with clustered data: adjusting for the cluster-mean of the exposure and the cluster-size, respectively, in addition to the other predictors of interest. Both approaches effectively adjust on cluster-level summaries of the data and can explain part of the between-cluster variability. As a result they can provide more accurate marginal predictions compared to those obtained from a model that ignores CBC/ICS. This is important because even for clusters whose estimated random effects are unknown because they were not part of the development sample, and thus only marginal predictions can be readily obtained, adjusting for ICS/CBC when present can improve the accuracy of marginal predictions. In particular, the higher the proportion of the between-cluster-variability explained the higher the accuracy gained by accounting for CBC/ICS. When either the degree of clustering in the data is small (e.g. ICC < 0.1) and/or the proportion of the between cluster-variability explained by *N* and/or $\overline{R}$ is small, accounting for CBC/ICS will offer minimal improvement in the accuracy of marginal predictions. On the other hand, ignoring the presence of CBC/ICS has no impact on the accuracy of conditional predictions.

The sample size for the application of the methods described in this paper for the purposes of risk prediction should abide by the rules for the required sample size, events per variable (EPV) and numbers of clusters for risk prediction as outlined by previous authors [11, 29]. A sufficiently large number of EPV and a large number of clusters would ensure that both the fixed and random effects are estimated well, and hence the predictions are reliable. So, depending on the prognostic strength of the model, the EPV should be large-enough to avoid model-overfitting (EPV at least 10 as a rough guide) [29] and the number of clusters should not be too small [18] (> 5, but close to 50 when possible, in order for the random effects and coefficients of cluster-level variables to be estimated well). In Supplementary Material 1 we present a simulation example where the number of clusters was 15, the average cluster size 50 and the prevalence 15%, all smaller than the values considered in the main simulation scenarios, showing that the methods described in this paper also perform well in this scenario (Figure S3).

It should be noted that the requirement for the availability of information for the whole cluster may sometimes pose challenges in the applicability of these two approaches. For example, to obtain predictions for a single patient who belongs to a hospital that is not part of the development sample, we would need to know the characteristics of the patient but also the predictor values (or the size) of that entire hospital, information that may not be readily available. In the complete absence of such cluster-level information, a reasonable approach would be to assume an ‘average’ exposure level (or size) for the hospital. In this article we focused on scenarios where there is one level of clustering, e.g. patients within hospitals. In principle, more levels of clustering may be present, for example when patients are treated by specific surgeons, who operate within specific hospitals. In such scenarios, the clustering in GLMM can be readily handled by using additional random effects, although care should be taken in assessing ICS/CBS in the levels of these more complex structures and obtaining marginal and conditional predictions from a GLMM.

## Conclusion

To our knowledge, ICS and CBC have been generally overlooked in the context of risk prediction. Departures from the usual relationship between marginal and conditional coefficients when developing risk models with clustered data in practice have been reported in the literature [27]. Even though such departures can be symptoms of CBC, the issue has been routinely ignored in the process of developing a risk model. Using simple diagnostic procedures to detect and handle these complications is important and should be part of the model-development process when developing models for clustered data. Any source of cluster-level information that reduces the unexplained between-cluster variability should be utilised to obtain more accurate marginal predictions.

## Supplementary Information

**Additional file 1.**

**Additional file 2.**

## Data Availability

The data that support the findings of this study are available from the Global Study Tumour Study Group (GSTSG) but restrictions apply to the availability of these data, which were used under license for the current study, and so are not publicly available. Data are however available from the authors upon reasonable request and with permission of the GSTSG. Software code (R) written for the simulation studies is available from the corresponding author on reasonable request.

## Abbreviations

CBC: Confounding by Cluster
CML: Conditional Maximum Likelihood
GEE: Generalised Estimating Equations
GLMM: Generalised Linear Mixed Model
ICC: Intra-cluster Correlation Coefficient
ICS: Informative Cluster Size
IEE: Independence Estimating Equations
MLE: Maximum Likelihood Estimation
SPT: Secondary Prostate Tumour

## References

[1] Berlin JA, Kimmel SE, Ten Have TR (1999). An empirical comparison of several clustered data approaches under confounding due to cluster effects in the analysis of complications of coronary angioplasty. Biometrics.

[2] Have TRT, Ratcliffe SJ, Reboussin BA, Miller ME (2004). Deviations from the population-averaged versus cluster-specific relationship for clustered binary data. Stat Methods Med Res.

[3] Localio AR, Berlin JA, Have TRT (2002). Confounding due to cluster in multicenter studies—causes and cures. Health Serv Outcome Res Methodol.

[4] Williamson JM, Datta S, Satten GA (2003). Marginal analyses of clustered data when cluster size is informative. Biometrics.

[5] Hoffman EB, Sen PK, Weinberg CR (2001). Within-cluster resampling. Biometrika.

[6] Chen Z, Zhang B, Albert PS (2011). A joint modeling approach to data with informative cluster size: robustness to the cluster size model. Stat Med.

[7] Anderson GL (1994). A cautionary note on inference for marginal regression models with longitudinal data and general correlated response data AU - Sullivan pepe, Margaret. Communications in Statistics - Simulation and Computation.

[8] Ten Have TR, Landis JR, Weaver SL (1995). Association models for periodontal disease progression: a comparison of methods for clustered binary data. Stat Med.

[9] Seaman S, Pavlou M, Copas A (2014). Review of methods for handling confounding by cluster and informative cluster size in clustered data. Stat Med.

[10] Bouwmeester W, Twisk JW, Kappen TH (2013). Prediction models for clustered data: comparison of a random intercept and standard regression model. BMC Med Res Methodol.

[11] Wynants L, Bouwmeester W, Moons KGM (2015). A simulation study of sample size demonstrated the importance of the number of events per variable to develop prediction models in clustered data. J Clin Epidemiol.

[12] Authors/Task Force m, Windecker S, Kolh P, et al. (2014). 2014 ESC/EACTS guidelines on myocardial revascularization: the Task Force on myocardial revascularization of the European Society of Cardiology (ESC) and the European Association for Cardio-Thoracic Surgery (EACTS) developed with the special contribution of the European Association of Percutaneous Cardiovascular Interventions (EAPCI). Eur Heart J.

[13] Pavlou M, Ambler G, Seaman S (2015). A note on obtaining correct marginal predictions from a random intercepts model for binary outcomes. BMC Med Res Methodol.

[14] Falconieri N, Van Calster B, Timmerman D (2020). Developing risk models for multicenter data using standard logistic regression produced suboptimal predictions: a simulation study. Biom J.

[15] Eldridge SM, Ukoumunne OC, Carlin JB (2009). The intra-cluster correlation coefficient in cluster randomized trials: a review of definitions. Int Stat Rev.

[16] Skrondal A, Rabe-Hesketh S (2009). Prediction in multilevel generalized linear models. J R Stat Soc.

[17] Zeger SL, Liang KY, Albert PS (1988). Models for longitudinal data: a generalized estimating equation approach. Biometrics.

[18] Wynants L, Vergouwe Y, Van Huffel S (2018). Does ignoring clustering in multicenter data influence the performance of prediction models? A simulation study. Stat Method Med Res.

[19] Neuhaus JM, Kalbfleisch JD, Hauck WW (1991). A comparison of cluster-specific and population-averaged approaches for analyzing correlated binary data. Int Stat Rev Revue Internationale de Statistique.

[20] Brumback BA, Dailey AB, Brumback LC, Livingston MD, He Z (2010). Adjusting for confounding by cluster using generalized linear mixed models. Stat Amp Probability Let.

[21] Nevalainen J, Datta S, Oja H (2014). Inference on the marginal distribution of clustered data with informative cluster size. Stat Pap (Berl).

[22] O’Neill D, Nicholas O, Gale Chris P (2017). Total Center percutaneous coronary intervention volume and 30-day mortality. Circulation.

[23] Huang Y, Leroux B (2011). Informative cluster sizes for subcluster-level covariates and weighted generalized estimating equations. Biometrics.

[24] Dunson DB, Chen Z, Harry J (2003). A Bayesian approach for joint modeling of cluster size and subunit-specific outcomes. Biometrics.

[25] Benhin E, Rao JNK, Scott AJ (2005). Mean estimating equation approach to analysing cluster-correlated data with nonignorable cluster sizes. Biometrika.

[26] Steyerberg EW, Vickers AJ, Cook NR, Gerds T, Gonen M, Obuchowski N, Pencina MJ, Kattan MW (2010). Assessing the performance of prediction models a framework for traditional and novel measures. Epidemiology.

[27] Wynants L (2016). Clinical risk prediction models based on multicenter data : methods for model development and validation. PhD thesis.

[28] Wynants L, Kent DM, Timmerman D, Lundquist CM, van Calster B (2019). Untapped potential of multicenter studies: a review of cardiovascular risk prediction models revealed inappropriate analyses and wide variation in reporting. Diagnostic Prognostic Res.

[29] Riley RD, Snell KI, Ensor J (2019). Minimum sample size for developing a multivariable prediction model: PART II - binary and time-to-event outcomes.

