# Supplementary material for: Risk prediction in multicentre studies when there is confounding by cluster or informative cluster size
Source: BMC Med Res Methodol. 2021 Jul 4;21:135. doi: 10.1186/s12874-021-01321-x (PMC8254921; doi:10.1186/s12874-021-01321-x)
Supplement: Supplementary file 1 — Additional file 1. [file 12874_2021_1321_MOESM1_ESM.docx]

Supplementary Material for the article
‘Risk prediction in multicentre studies when there is confounding by cluster or informative cluster size’

# Practical example demonstrating the effect of CBC on the estimation of regression coefficients for different analysis methods

The effect of CBC on the regression coefficient is illustrated using one dataset, simulated in the same mechanism as in Section 3 of the main paper. The ICC is chosen to be 0.2, and the values of the 6 explanatory variables were set to 1. The exposure variable is variable $X_{6}.$

We consider two scenarios were the random intercept terms for the clusters are either
a) independent of the exposure (no-CBC)
b) associated with the exposure (CBC)

For the two scenarios above we use IEE and GLMM to fit both the Basic model which ignores CBC (‘Basic’) and the model that adjusts for the cluster-mean of the exposure and estimates the within-cluster effect of the exposure. We present coefficient estimates for the Basic model and the model that adjusts for$\bar{X}_{6}$, using IEE, GLMM. We also present coefficient estimates from the use of Conditional Maximum Likelihood.

**Scenario 1: No confounding by cluster**

| **Basic Model** | | | |  | **Adjust for the cluster mean of X6** | | | |
| --- | --- | --- | --- | --- | --- | --- | --- | --- |
|  | **IEE** | **GLMM** | **CL** |  |  | **IEE** | **GLMM** | **CL** |
| **x1** | 0.96 | 1.09 | 1.09 |  | **x1** | 0.96 | 1.09 | 1.09 |
| **x2** | 0.88 | 0.98 | 0.98 |  | **x2** | 0.88 | 0.98 | 0.98 |
| **x3** | 0.89 | 1.02 | 1.02 |  | **x3** | 0.89 | 1.02 | 1.02 |
| **x4** | 0.96 | 1.07 | 1.07 |  | **x4** | 0.96 | 1.07 | 1.07 |
| **x5** | 0.85 | 0.96 | 0.96 |  | **x5** | 0.85 | 0.96 | 0.96 |
| **x6** | 0.89 | 1.01 | 1.01 |  | **x6w** | 0.89 | 1.01 | 1.01 |
| **ICC** |  | **0.17** |  |  | **ICC** |  | **0.17** |  |

We observed that the marginal regression coefficients are attenuated towards zero compared to the conditional coefficients, as expected. The within-cluster effect of $X_{6}$ remains the same regardless of whether we adjust for $\bar{X}_{6}$or not. The ICC is estimated to be 0.17 in both models, as the adjustment variable $\bar{X}_{6}$ does not have any predictive value in the absence of CBC.

**Scenario 2: Confounding by cluster**

| **Basic Model** | | | |  | **Adjust for the cluster mean of X6** | | | |
| --- | --- | --- | --- | --- | --- | --- | --- | --- |
|  | **IEE** | **GLMM** | **CL** |  |  | **IEE** | **GLMM** | **CL** |
| **x1** | 1.00 | 1.12 | 1.12 |  | **x1** | 0.96 | 1.09 | 1.12 |
| **x2** | 0.88 | 0.96 | 0.96 |  | **x2** | 0.88 | 0.98 | 0.96 |
| **x3** | 0.91 | 1.03 | 1.03 |  | **x3** | 0.89 | 1.02 | 1.03 |
| **x4** | 0.91 | 1.01 | 1.01 |  | **x4** | 0.96 | 1.07 | 1.01 |
| **x5** | 0.87 | 0.97 | 0.97 |  | **x5** | 0.85 | 0.96 | 0.97 |
| **x6** | 1.34 | 1.04 | 1.02 |  | **x6w** | 0.89 | 1.01 | 1.02 |
| **ICC** |  | 017 |  |  | **ICC** |  | **0.08** |  |

We observe that the expected relationship between marginal and conditional coefficients is distorted for the exposure variable $X_{6}$($\hat{b}_{6}^{IEE}=1.34>$ $\hat{b}_{6}^{GLMM}=1.04$) when the Basic model is used. After adjusting for the cluster mean of the exposure, the usual relationship is restored. The ICC is estimated to be 0.17 in the Basic model but is reduced to 0.08 for the model that adjusts for $\bar{X}_{6},$since this variable now carries a predictive value thus reducing the previously unexplained between-cluster variability in the outcome.

# Additional Simulation Results

## Scenario 4: ICS and CBC through related or unrelated mechanisms


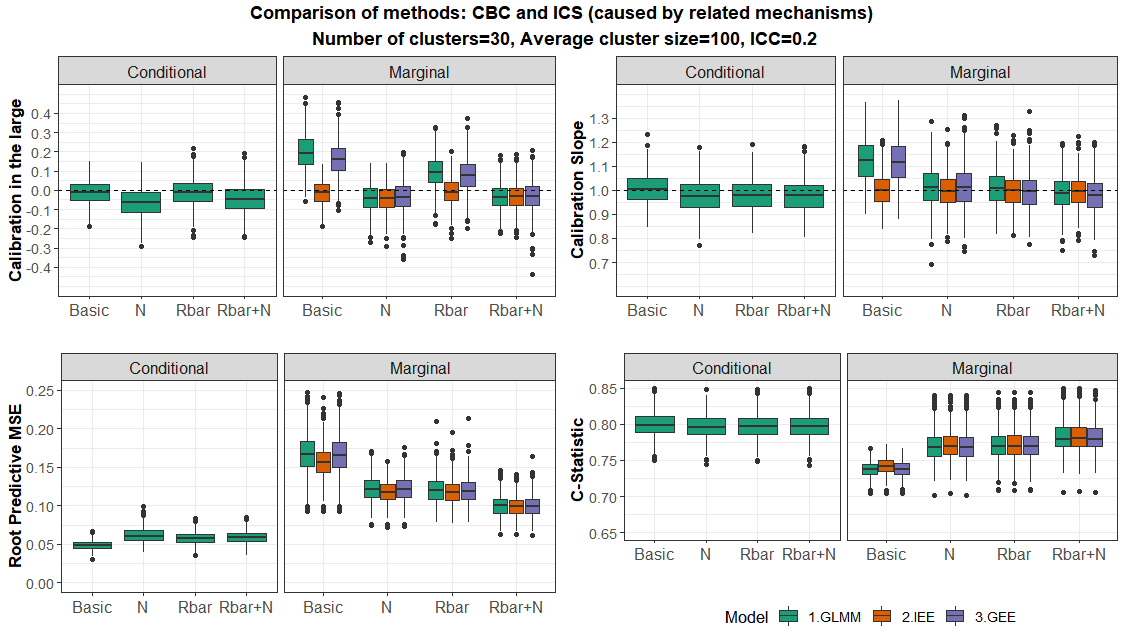


Figure S1: ICS and CBC arising through a related mechanism ($\rho_{vw}=0.5$). Comparison of Basic models (‘Basic’) and models adjusting for $N$ and/or  $\bar{R}.$Conditional (left Panel) and Marginal (right panel) predictions are shown. Performance measures from top left to bottom right: Calibration Intercept, Calibration slope, RPMSE and C-statistic.


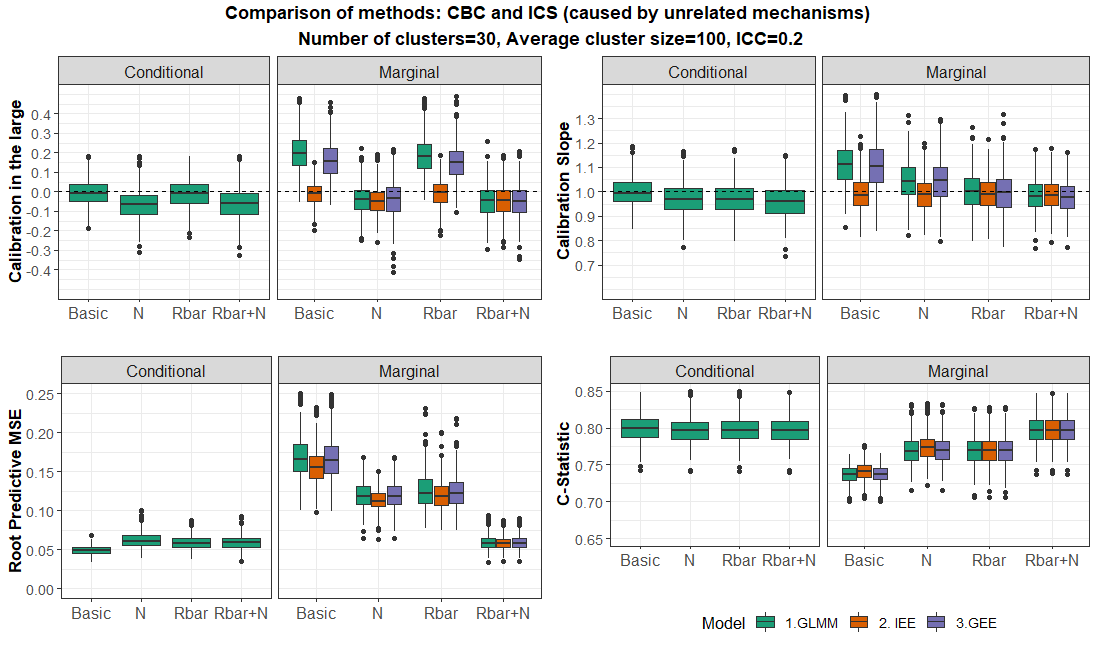


Figure S2: ICS and CBC arising through a related mechanism ($\rho_{vw}=0$). Comparison of Basic models (‘Basic’) and models adjusting for $N$ and/or  $\bar{R}.$ Conditional (left Panel) and Marginal (right panel) predictions are shown. Performance measures from top left to bottom right: Calibration Intercept, Calibration slope, RPMSE and C-statistic.

## Small sample size: Simulation example for a scenario with smaller data: number of clusters =15, average cluster size=50 and prevalence=15%


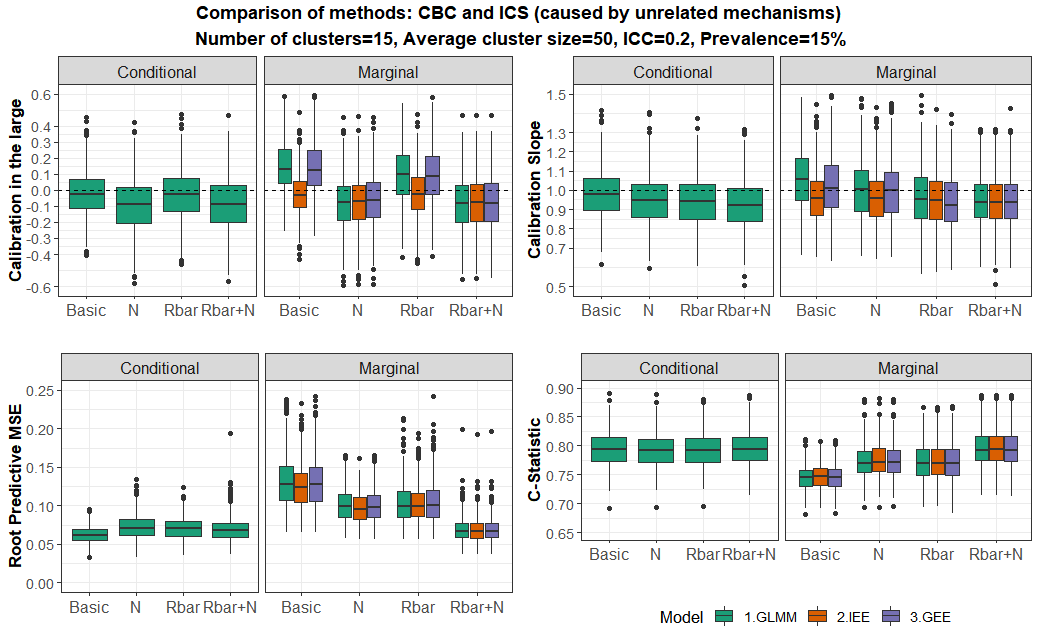


Figure S3: ICS and CBC arising through a related mechanism ($\rho_{vw}=0$) when the number of clusters is 15, the average cluster size is560 and the prevalence=15%. Comparison of Basic models (‘Basic’) and models adjusting for $N$ and/or  $\bar{R}.$ Conditional (left Panel) and Marginal (right panel) predictions are shown. Performance measures from top left to bottom right: Calibration Intercept, Calibration slope, RPMSE and C-statistic.

## Additional simulation results for ICC=0.1.

The following Figures are analogous to the ones presented in the main article, albeit for Intra-cluster correlation coefficient, ICC=0.1.


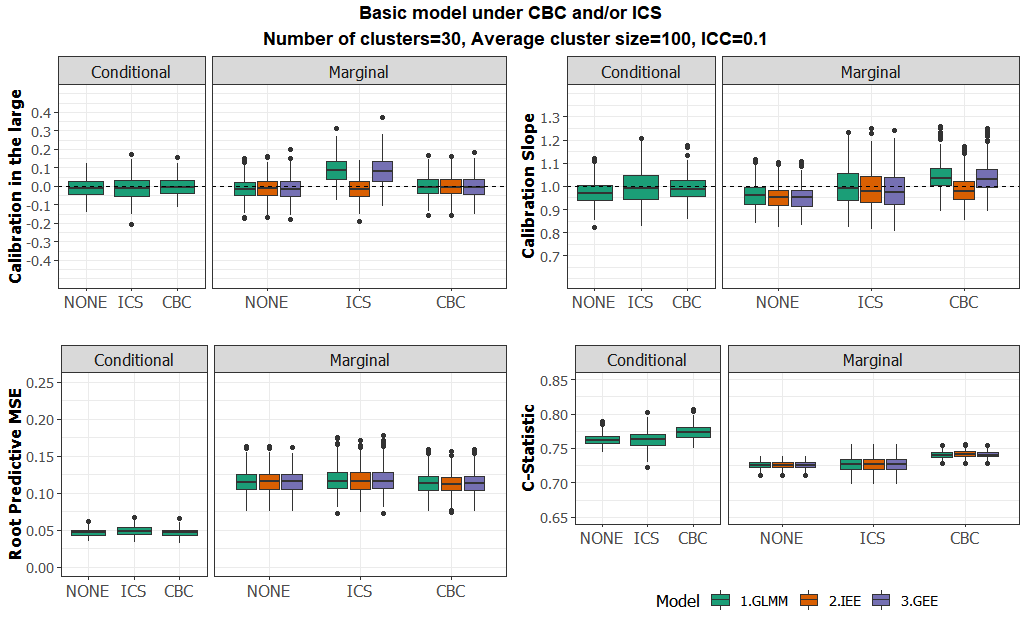


Figure S4: Comparison of the different fitting methods (GLMM, IEE, GEE with exchangeable correlation) for the Basic model, under different assumptions about the presence of CBC and ICS. ICC=0.1. Performance measures from top left to bottom right: Calibration in the large, Calibration slope, Root Predictive MSE and C-statistic. Conditional (left Panel) and Marginal (right panel) predictions are show for each measure.


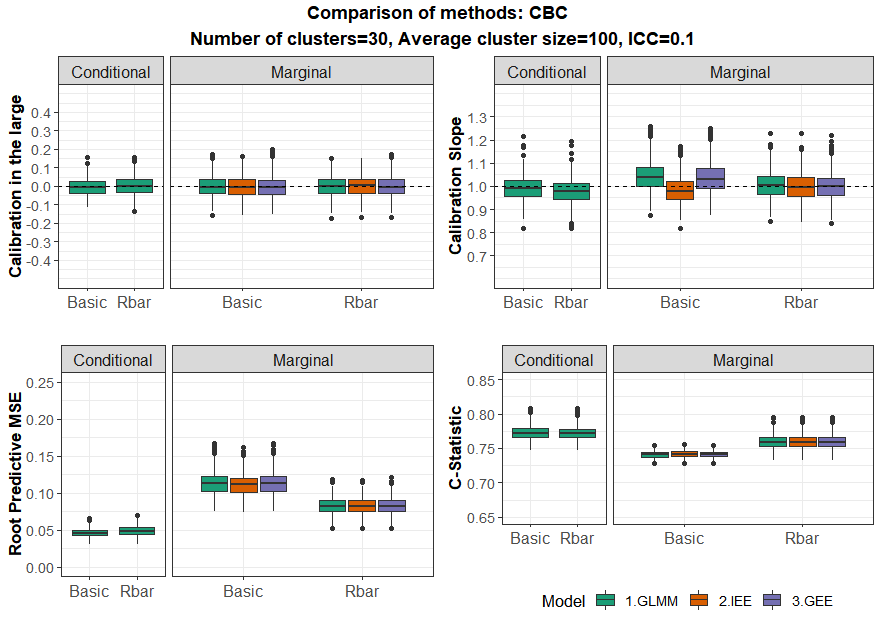


Figure S5: Confounding by Cluster (CBC). Comparison of Basic models (‘Basic’) and models also adjusting for the cluster mean of the exposure ($\bar{R}$) fitted by three methods (GLMM, IEE, and GEE with exchangeable correlation.). ICC=0.1. Performance measures from top left to bottom right: Calibration Intercept, Calibration slope, RPMSE and C-statistic. Conditional (left Panel) and Marginal (right panel) predictions are shown.


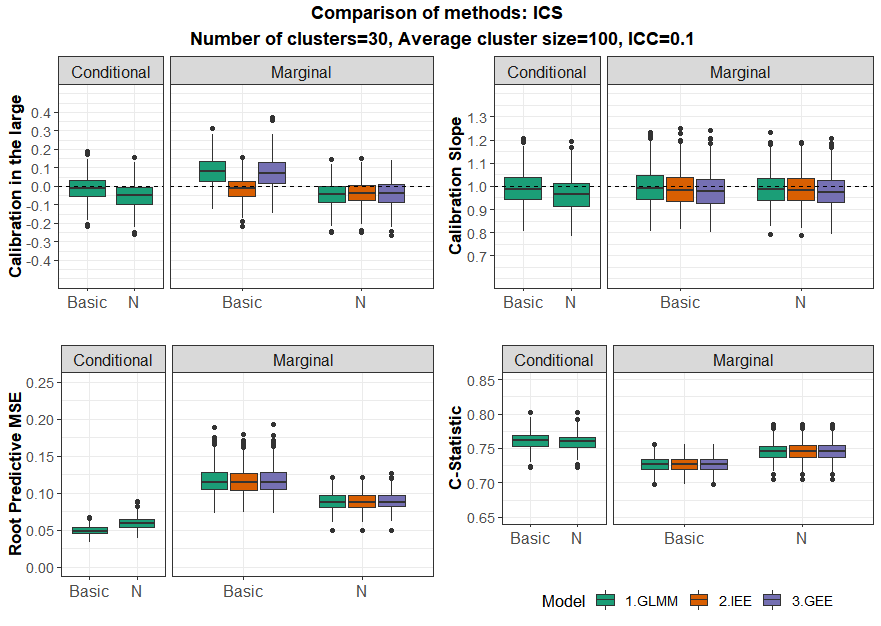


Figure S6: Informative Cluster size (ICS). Comparison of Basic models (‘Basic’) and models adjusting for the cluster size (‘N’) fitted by three Methods. ICC=0.1. Conditional (left Panel) and Marginal (right panel) predictions are shown. Performance measures from top left to bottom right: Calibration Intercept, Calibration slope, RPMSE and C-statistic.
